# *Plasmodium vivax* vaccine: What is the best way to go?

**DOI:** 10.3389/fimmu.2022.910236

**Published:** 2023-01-16

**Authors:** Gisele Tatiane Soares da Veiga, Matheus Ribeiro Moriggi, Jean Franciesco Vettorazzi, Marcelo Müller-Santos, Letusa Albrecht

**Affiliations:** ^1^ Laboratory of Apicomplexan Parasites Research, Carlos Chagas Institute, Oswaldo Cruz Foundation (FIOCRUZ), Curitiba, Brazil; ^2^ Nitrogen Fixation Laboratory, Department of Biochemistry and Molecular Biology, Federal University of Paraná (UFPR), Curitiba, Brazil; ^3^ Biomedical Sciences Course, Educational Union of Cascavel (UNIVEL), Cascavel, Brazil

**Keywords:** malaria, *Plasmodium vivax*, vaccine, subunit, vaccine candidates

## Abstract

Malaria is one of the most devastating human infectious diseases caused by *Plasmodium* spp. parasites. A search for an effective and safe vaccine is the main challenge for its eradication. *Plasmodium vivax* is the second most prevalent *Plasmodium* species and the most geographically distributed parasite and has been neglected for decades. This has a massive gap in knowledge and consequently in the development of vaccines. The most significant difficulties in obtaining a vaccine against *P. vivax* are the high genetic diversity and the extremely complex life cycle. Due to its complexity, studies have evaluated *P. vivax* antigens from different stages as potential targets for an effective vaccine. Therefore, the main vaccine candidates are grouped into preerythrocytic stage vaccines, blood-stage vaccines, and transmission-blocking vaccines. This review aims to support future investigations by presenting the main findings of vivax malaria vaccines to date. There are only a few *P. vivax* vaccines in clinical trials, and thus far, the best protective efficacy was a vaccine formulated with synthetic peptide from a circumsporozoite protein and Montanide ISA-51 as an adjuvant with 54.5% efficacy in a phase IIa study. In addition, the majority of *P. vivax* antigen candidates are polymorphic, induce strain-specific and heterogeneous immunity and provide only partial protection. Nevertheless, immunization with recombinant proteins and multiantigen vaccines have shown promising results and have emerged as excellent strategies. However, more studies are necessary to assess the ideal vaccine combination and test it in clinical trials. Developing a safe and effective vaccine against vivax malaria is essential for controlling and eliminating the disease. Therefore, it is necessary to determine what is already known to propose and identify new candidates.

## 1 Introduction

Malaria has a devastating impact on people’s quality of life, mortality, and morbidity. In 2020, it caused more than 241 million cases worldwide and killed approximately 627 thousand people (1). It occurs in several tropical and subtropical regions, with a high incidence in sub-Saharan Africa, Southeast Asia, the Eastern Mediterranean, the Western Pacific, and the Americas (1). It is caused by *Plasmodium* spp. And transmitted during the blood meals of infected females of *Anopheles* spp. Mosquitoes. Seven species of *Plasmodium* affect humans: *P. falciparum*, *P. vivax*, *P. malariae*, *P. ovale*, *P. knowlesi*, *P. cynomolgy* and *P. simium*, of which *P. falciparum* and *P. vivax* are responsible for approximately 99% of malaria cases. Although *P. falciparum* is the deadliest parasite, *P. vivax* is the most geographically spread (1).

*P. vivax* was neglected for decades because of the belief that its infection causes milder disease (2). However, recent studies reported severe cases caused by vivax malaria, such as severe anemia, cerebral malaria, thrombocytopenia, and acute respiratory syndrome (3–9). Studies have shown that *P. vivax* has the ability to promote cytoadherence to the host’s endothelium and form rosettes (10–12), features frequently associated with severe *P. falciparum* malaria (13, 14). Nevertheless, due to the numerous challenges that this parasite poses, there are few vaccines under development (15).

The development of a safe and effective vaccine against malaria is the best and most relevant strategy for preventing, controlling, and eliminating malaria. However, we are still far from achieving this, especially for vivax malaria (16). The difficulty in finding the best immunogen combined in a formulation that induces protection is the main challenge in vivax vaccines. The majority of experimental vaccines against *P. vivax* contain a recombinant *P. vivax* protein with an adjuvant. In addition, different technologies have been used in an attempt to improve malaria vaccine efficacy, such as recombinant viral vectors, virus-like particles (VLPs), DNA plasmids, long synthetic peptides (LSPs) and irradiation-attenuated parasites. However, they all failed to induce a strong protective effect. For *P. falciparum* malaria prevention, a vaccine called Mosquirix (RTS, S) was recently approved for children (ClinicalTrials.gov number, NCT00380393). The RTS/S is a subunit vaccine that targets the central repeats and the C-terminal of the *P. falciparum* circumsporozoite surface protein (PfCSP), including T-cell epitopes (T) fused to hepatitis B surface antigen (HBsAg) (S), mixed with native HBsAg (S), formulated with the AS01 adjuvant. The vaccine efficacy against clinical malaria over 4 years of follow-up was estimated at 25.9% in infants and at 36,3% in children, but as the age and time of vaccination advance, the estimative drops to low levels (17, 18). Over 7 years of follow-up, the efficacy was estimated at 4.4% (19).

R21/MatrixM is a vaccine candidate in the approval process (ClinicalTrials.gov number, NCT03896724) with promising results of 77% efficacy in phase III studies (20). However, for *P. vivax*, the scenario is meagre. To this parasite, there are only three antigens in the initial clinical phases, presented in **Table 1**, which confer only partial protection and few candidates in preclinical trials (28). The antigens used in malaria vaccine formulations have a high polymorphism, which induces a strain-specific response representing a significant challenge to vaccine development (29). Many of the antigens evaluated in vivax malaria vaccines were initially discovered for *P. falciparum* and are not necessarily the best antigens in a vivax vaccine. To date, it has not been possible to establish *P. vivax* long-term *in vitro* culture, which makes antigen discovery more challenging. Thus, some studies seek to identify parasite proteins *in silico*, whereas features such as antigenicity and immunogenicity are evaluated, and antigens against different stages of the paras’te’s life cycle combined with different adjuvants and immunization schedules have been tested (30–33). These vaccine candidates are categorized as preerythrocytic stage vaccines, blood-stage vaccines, and transmission-blocking vaccines (34).

**Table 1:** *Plasmodium vivax* vaccines in clinical trials.

Candidate	Phase	Key findings	Clinical Trial Number	References
Preerythrocytic stage vaccines				
VMP001	1/2a	Recombinant PvCSP with adjuvant AS01B. Reduction of parasitemia, but low efficacy.	NCT01157897	(21)
Peptides N R&C	1b/2	PvCSP derived from long synthetic peptides (LSP) with Montanide ISA 720 and 51. Long-lasting antibody response, with 36.6% efficacy in naive volunteers.	NCT0108184	(22, 23)
PvRAS	1/2a	*P. vivax* irradiated sporozoite. Poor cellular response and 42% efficacy.	NCT01082341	(24)
Blood-stage vaccines				
ChAd63-MVA-PvDBPII	1a/2a	Heterologous *prime-boost* regimen with recombinant viral vectors ChAd63-MVA-PvDBPII. Induction of antibodies that inhibit interaction with reticulocytes, humoral and cellular response, 50% of strain-transcendent immunity.	NCT01816113	(25)
PvDBPII-GLA-SE	1	Recombinant PvDPBII with GLA-SE adjuvant. High production of specific antibodies can inhibit interaction with reticulocytes and strain-transcendent response.	CTRI/2016/09/007289	(26)
Transmission-blocking vaccines				
Pvs25	1	Recombinant Pvs25 with Montanide ISA 51 adjuvants. Good induction of antibodies and 30% reduction in infected mosquitoes. High reactogenicity.	NCT00295581	(27)

**Figure 1:**
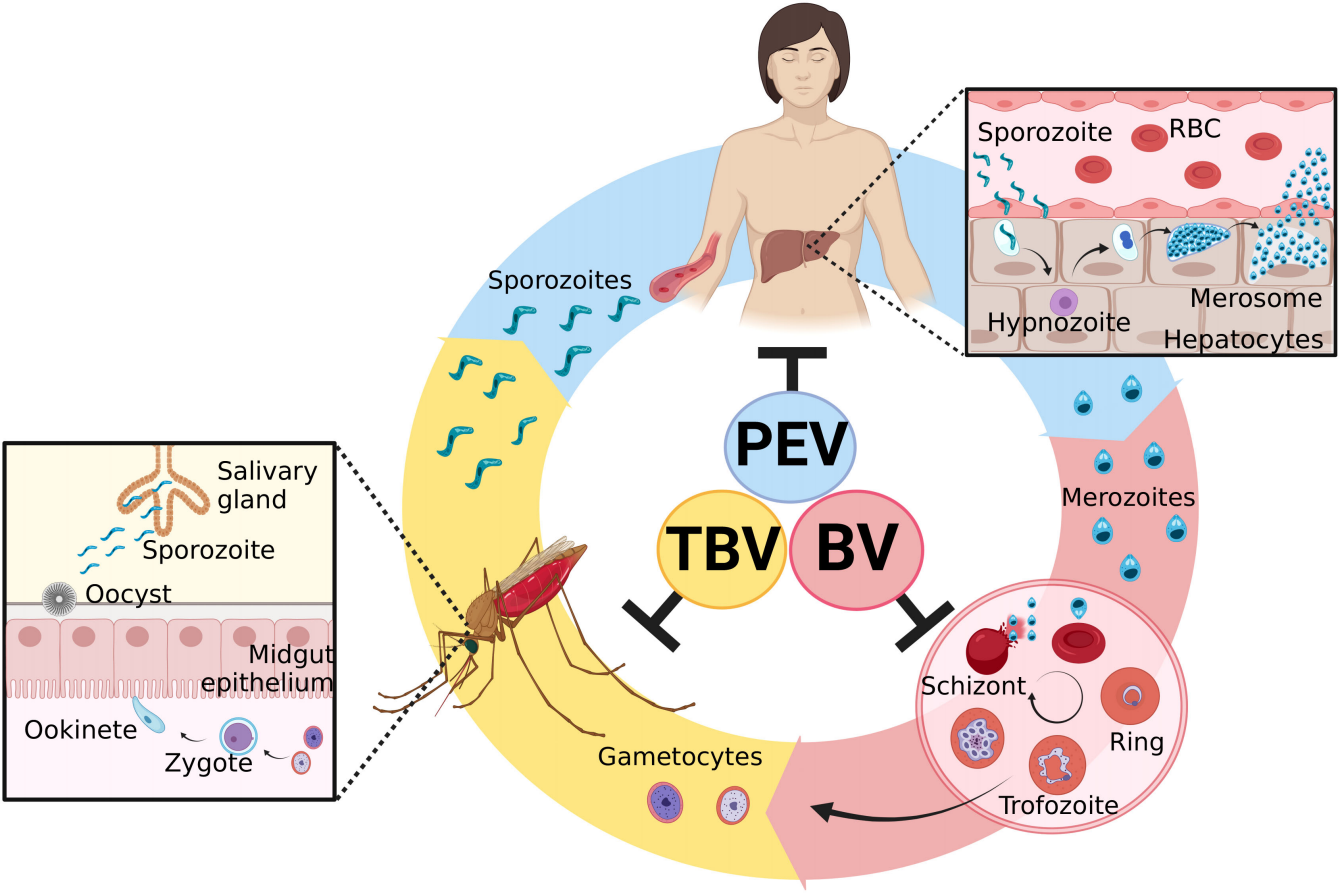
*Plasmodium vivax* life cycle and targets of malaria vaccines. Sporozoites are inoculated in the host skin during the blood repast of female *Anopheles* spp. They move to the liver by the circulatory system and invade hepatocytes. PEVs (preerythrocytic vaccines) are designed to block this step. Inside the liver cells, morphological changes occur in the parasite and might lead to the development of hypnozoites, the latent hepatic form. After its activation, the liver schizont is formed, followed by the merosome. When it ruptures, it releases merozoites into the blood circulation. BV (blood vaccines) are designed to prevent the invasion of reticulocytes by merozoites. After erythrocyte invasion, new forms are generated, including male and female gametocytes, which stay in the blood. If a new blood repast happens, these forms are ingested by mosquitoes, where the sexual cycle takes place. The gametocytes fertilize, giving rise to the zygote and then the ookinete. The ookinete invades the midgut epithelium of the mosquito and originates the oocyst. When it ruptures, it releases sporozoites, which migrate to the salivary gland and can restart the cycle. TBV (transmission-blocking vaccines) are designed to interrupt this development to reduce transmission. Created with BioRender.com.

The parasite’s life cycle begins in the vertebrate host during the blood repast of the infected *Anopheles* spp. female (35). The sporozoites along with the salivary fluid are inoculated into the dermal tissues. Into skin, sporozoites acquire motility and are able to penetrate the blood vessels and start stimulating a host immune response (36, 37). After that, sporozoites move through the blood system to the liver, invading hepatocytes (38, 39). Here, a preerythrocytic vaccine could interrupt this interaction or its subsequent intracellular development (40–42). In liver cells, a series of morphological changes occur. It can generate hypnozoites, the dormant stage, which can remain in the liver for many years and cause numerous recurrences of the disease (43, 44). After modifications in the hepatic phase, merozoites are released into the bloodstream (45), and different from other *Plasmodium* species, in *P. vivax* infection, the parasite selectively invades only reticulocytes, which represents less than 2% of all erythrocytes in human blood, developing low parasitemia (46, 47). Here, blood-stage vaccines attempt to stop the invasion of blood cells by merozoites or stop their development into red blood cells, preventing the onset of symptoms (40–42). Inside the cells, the parasite quickly undergoes further modifications, giving rise to gametocytes, which are rapidly transmitted to mosquitoes during a new blood meal (48, 49). In the invertebrate host, gametocytes form gametes that will be fertilized to form tokineteete. This life form invades the epithelium of the insects’ midgut, forming the oocyst, which releases sporozoites in the hemolymph. Sporozoites migrate to the salivary gland and can continue the cycle (42). Then, transmission-blocking vaccines aim to stop the fusion of gametes and subsequent development in the invertebrate host (40–42). The cycle and the targets of malaria vaccines are illustrated in **Figure 1**.

This review summarizes and discusses the main approaches, the results, and challenges related to the development of vaccines against *P. vivax*. Our main objective is to elucidate and clarify the situation of vivax malaria vaccines, trying to investigate the best way to forward further studies.

## 2 Insights into the immune response in *Plasmodium vivax* infection

Immunity to *Plasmodium* is complex, and many aspects remain poorly understood. In a natural immune response, short-term protection is induced, as individuals are often reinfected. However, repeated exposure to *Plasmodium* can induce immunity, supporting the potential of vaccines (50). The quality of the immune response is highly variable and will depend on several factors, as it increases with age, prior exposure, and transmission intensity (51). Both innate and adaptive responses are important for the development of acquired immunity (52).

Dendritic cells (DC) recognize *Plasmodium* pathogen-associated molecular patterns (PAMPs), such as hemozoin, immunostimulatory nucleic acid motifs, and glycosylphosphatidylinositol anchors (GPI), leading to activation of T and B lymphocytes, natural killer (NK) cells and macrophages (53). Immune cells are induced to produce interferon-y (IFN-γ). This cytokine plays an important role in the control of parasitemia both in the hepatic and blood stages (54, 55). IFN-γ activates CD8+ T cells, B cells, and macrophages (56, 57). It induces isotype switching, leading to the production of cytophilic antibodies, which can bind to merozoites, block reticulocyte invasion, opsonize the parasite and promote phagocytosis by macrophages (54). IFN-γ can induce the expression of MHC class I in reticulocytes, leading to the elimination of infected cells by T cells (58). Additionally, this cytokine is important in the differentiation of atypical memory B cells (MBCs) into plasma cells (59).

In the preerythrocytic stage, the cell response predominates since sporozoite surface antigens can be recognized by MHC class I of CD8+ T cells, leading to the activation and proliferation of these cells. On the other hand, in the blood stage, the humoral response prevails (28). The antibody response, especially IgG3 and IgG1 cytophilic antibodies, can neutralize sporozoites and inhibit invasion of hepatocytes, opsonized merozoites and block reticulocyte invasion, activate cell-mediated death and are associated with protection against clinical disease (28). However, there is still no consensus on the longevity of humoral responses during malaria, since immunity is antigen specific. However, studies report that it rapidly declines after parasite clearance (50).

The comprehension of the association between protective immunity and the longevity of antibodies and MBCs is valuable for future vaccine development since MBCs are major contributors to antibody production. However, the persistence of specific MBCs to *P. vivax* antigens remains unclear (50). Several studies have investigated the relationship between the induction of specific antibodies and specific-MBC responses. In general, an increase in the number of plasma cells is observed during *P. vivax* acute infection, but the presence of long-lived MBCs was not consistently related to the antibody response (60–66).

The presence of antigen-specific MBCs was detectable at 6 months for PvAMA-1 and PvMSP-9 (66), 9 months for PvDBPII (60) and PvMSP1-19 (65), 12 months for PvMSP-8 (65), 3 years post-infection for PvDBPII (60) and PvRBP1a (64), and even for up to 4 years post-infection for PvMSP-8 (65), suggesting a long-lasting immune response and potential for vaccines. In addition, *P. vivax* acute infection is characterized by an increase in the frequencies of activated and atypical MBC populations (60, 62–64). The increase in these cells is a consequence of augmented systemic IFN-γ production and multiple stimuli, such as TLR7/8 and IL-21 (59). However, the exact function of atypical MBCs and their relation to previous infection remain uncertain. Thus, more studies are necessary to understand its role in *Plasmodium vivax* infection.

## 3 Preerythrocytic stage vaccine

**Figure 2:**
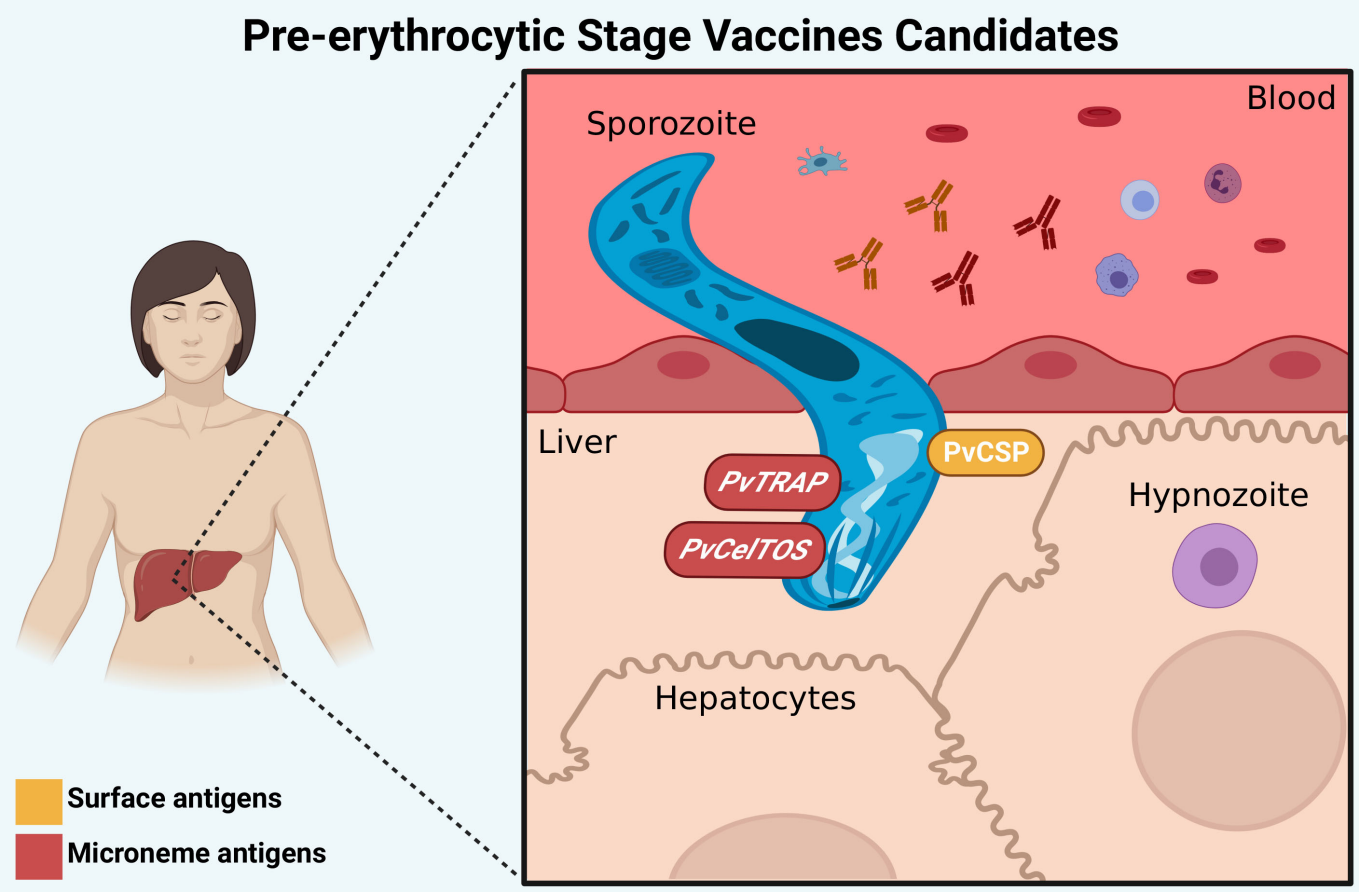
Preerythrocytic stage vaccine targets. The main candidates for PEV are PvCSP (surface antigen), PvTRAP and PvCelTOS (microneme antigens), which have a role in the invasion process of hepatocytes. Antibodies against these proteins (represented by “Y shaped” on the figure) can interrupt the invasion of *P. vivax* to hepatocytes and the development of liver forms of the parasite. Created with BioRender.com.

Preerythrocytic vaccines target sporozoite antigens and could reduce infections by preventing hepatocyte invasion and establishing hypnozoites, which could achieve an anti-infection effect and decrease the risk of relapse and transmission (67). The main candidates for PEV are circumsporozoite surface protein (CSP) and thrombospondin‐related anonymous protein (TRAP) antigens (**Figure 2** **)**. It is important to note that the presence of hypnozoites in the liver is a major challenge to therapy development; thus, identifying new antigens derived from the dormant stage could be a strategy for vaccine development.

### 3.1 PvRAS

One of the vivax malaria vaccines tested in humans is the *P. vivax* radiation-attenuated sporozoites (PvRAS) vaccine, whereas sporozoites were inoculated into humans by mosquito bites. After immunization, individuals were challenged with live sporozoites, and 42% of them were protected (24). Neither IFN-γ nor total IgG was correlated with protection, although IgG1 and IgG3 antibodies to a synthetic PvCSP peptide were higher in protected individuals (24). These cytophilic antibodies might opsonize parasites by interacting with the Fc receptor on phagocytic cells. The possibility of using sporozoites in challenge models to test the efficacy of PEV was a great accomplishment; however, due to the lack of an *in vitro* culture for *P. vivax*, obtaining sporozoites is difficult. Therefore, the use of this method is limited to endemic regions, where blood from infected patients is available. Thus, the development of subunit vaccines has emerged as a strategy to overcome this limitation. The main candidates under study are described below.

### 3.2 PvCSP

The principal and the more advanced vaccine candidate at this stage employs the PvCSP. This protein consists of three main domains: the central region of tandem repeat sequences, of which three different variants are described, VK210, VK247, and *P. vivax*-like, in addition to the N- and C-terminal regions, which are two nonrepetitive conserved sequences (68–70). This protein is attached to the surface of the sporozoite and in early liver stages and is critical for sporozoite formation in the oocysts, invasion of mosquito salivary glands, and invasion of hepatocytes (71). Thus, a CSP-based PEV may induce a protective response by preventing sporozoites infection and inhibiting the establishment of hypnozoites (21).

The first vaccine study against PvCSP in humans was the Vivax Malaria Protein 001 (VMP001) associated with AS01B adjuvant. This vaccine consists of a chimeric protein that incorporates the amino and carboxy-terminal regions of the CSP and a short repeat sequence from the VK210 and VK247 parasite strains (72). The vaccine induced sterile protection in *Aotus nancymaae* and a high specific antibody response in a preclinical test when the antigen was combined with Montanide ISA 720 plus CpG 10104 adjuvants (73). However, phase I clinical trials did not induce sterile protection in humans despite providing a delay in parasitemia. In addition, the association of anti-VK210 repeat region antibodies and the prepatent period was observed, demonstrating that this region may improve strain-specific vaccine efficacy (21). Even if the *P. vivax* antigen was the same, vaccine formulations were made with different adjuvants, which could explain, at least in part, the different outcomes observed in those studies. Similarly, in the RTS, S vaccine, the adjuvant of choice was responsible for slightly different outcomes (18).

A trial was developed with another VMP001 formulation, defined as CSV-S, S, which is coexpressed as a hepatitis B fusion particle (74). The results showed that CSV-S, S and VMP001 were immunogenic and capable of inducing potent humoral and cellular responses in preclinical studies (74). The CSV-S, S, was better at inducing a higher antibody response in humans, indicating that more studies are necessary to investigate this candidate’s potential (75).

Salman et al. (76) tested a VLP platform using hepatitis B surface antigen to present PvCSP, called Rv21, a particle of CSV-S, S. This vaccine has the VK210 and VK247 sequences of PvCSP fused with hepatitis B viral particles and was expressed in the yeast *Pichia pastoris*. Rv21 vaccination combined with Matrix-M as an adjuvant showed highly lasting sterile protection against sporozoites in mice. Antibodies against both strains recognized the PvCSP on the surface of *P. vivax* sporozoites (76).

Different recombinant proteins, based on VK210, VK247, and *P. vivax*-like variants, were expressed in *Pichia pastoris* to obtain secreted proteins to immunize mice (77). One of the most promising antigens was a hybrid polypeptide called yPvCS-All epitopes, which contains epitopes of the three variants and a conserved C-terminal. This candidate was utilized in immunization with Poly (I:C). The results demonstrate a strong humoral and a low cellular response against all three repeat regions and the C-terminal. The vaccine reduced the liver parasite burden of C57BL/6 mice challenged with transgenic *P. berghei* sporozoites (77). Similarly, de Camargo et al. (78) developed a study involving two recombinant multiallelic proteins called yPvCSP-All_CT_ (containing only the C-terminal region) and yPvCSP-All_FL_ (containing the N- and C-terminal regions), both with the three central repeat regions of different PvCSP alleles. After immunization with the adjuvants, high levels of specific IgG were induced against the three variants. The protective efficacy of these vaccines, with Poly (I:C) as an adjuvant, was assessed with a homologous and a heterologous immunization system. Both vaccines protected mice against a PvCSP transgenic *P. vivax/P. berghei* parasite, provoking a significant delay in parasitemia (78).

Another promising study applied two recombinant proteins, consisting of the conserved N- and C-terminal regions flanking a truncated repeat region of either VK210/VK247, called rPvCS_127_, or VK247/VK210, called rPvCS_712_ (79). These proteins were recognized by monoclonal antibodies and the plasma of *P. vivax*-infected patients. Antibodies against these proteins recognize the native protein on the surface of *P. vivax* sporozoites (79). Both proteins were tested with naloxone (NLX), oligodeoxynucleotides (CpG-ODNs), and a saponin from the *Quillaja aponaria* tree (QS21), alone or in combination, in mice immunization (80). The results showed that both proteins and adjuvants could induce a high immune response, with antigen-specific humoral and cellular responses *in vivo*. When mice were immunized with PvCS_127_ or PvCS_712_, with all adjuvants, higher IgG2b, IgG2c, and IFN-γ levels were induced, indicating a Th1 response (80). However, when administered alone, the adjuvants CpG and QS21 induced higher avidity of antibodies than NLX. PvCS_712_ showed a marginally higher induction of humoral responses than PvCS_127_ (80).

Marques et al. (81) developed a vaccine derived from mumps’ viral nucleocapsid protein (NP). The PvCSP allelic variants (VK210, VK247, and *P. vivax*-like) were fused with the mumps virus NP in two different manners: NLP-CSP_R_ and NLP-CSP_CT_, in the absence or presence of the conserved C-terminal domain of PvCSP, respectively. After mouse immunization with these candidates and Poly (I:C), animals showed high IgG titers against all PvCSP variants, especially NLP-CSP_CT_. This antigen triggered a high titer of antibody against PvCSP repeated and nonrepeated regions, different from what was observed with the NLP-CSP_R_ (81). An increased number of antibody-secreting cells (ASCs) specific to NLP-CSP_CT_ was observed after the third immunization (81). After a mouse challenge with Pb/PvVK210 sporozoites, a decrease in parasitemia and 30% sterile protection in immunized animals were observed (81). Gimenez et al. (82) investigated the efficacy of NLP-CSP_CT_ and NLP-CSP_R_ vaccines against other strains (VK247 and *P. vivax*-like). Mice were immunized in a homologous prime-boost immunization system with three doses of the recombinant proteins in the presence of Poly (I:C) or Montanide ISA750 and challenged with transgenic parasites Pb/PvVK210, Pb/PvVK247 and Pb/PvCSP-like-G10. The study revealed a high induction of IgG against the three strains, which intensified after the second and third doses in all vaccine formulations, but a better response was observed with Montanide ISA750 (82). Protective efficacy was estimated by the time animals reached 1% parasitemia after challenge, and partial protection was observed in all vaccine formulation groups (82).

A platform based on Qβ VLP was used to test the efficacy of different epitopes from the VK210 and VK247 regions (83). The more immunogenic peptides were selected to be inserted into the Qβ particle for mouse vaccination. A tetramer, AGDR, within the nonamer repeat unit of VK210 is a target of neutralizing antibodies and was the only one to generate antibodies recognizing native PvCSP (83). It also conferred 100% protective efficacy against homologous challenge. In contrast, peptides derived from conserved regions (N- and C-terminal regions) could not confer protection (83). The Qβ-(AGDR) vaccine was tested with full-length and truncated PvCSP. These proteins alone conferred protection of 100% in the case of a truncated protein and 0% for the full-length protein. However, in combination with Qβ-(AGDR), the full-length protein showed an increase of 83% efficacy, while the truncated protein showed a decrease in efficacy (83).

Herrera et al. (84) determined a high antigenicity of malaria PvCSP–derived long synthetic peptides (LSPs) in exposed individuals and its immunogenicity in nonhuman primates. These polypeptides were designed and synthesized based on functional domains RI and RII (N and C peptides) or B and T-cell epitopes (R peptide) of the PvCSP (84). The vaccine formulation PvCSP-LSPs with Montanide ISA720 (85) or Montanide ISA51 (22) were safe and well tolerated in phase I clinical trials on malaria-naive volunteers in Colombia. All peptides could induce high specific antibodies and IFN-γ production at high doses, which remained for up to three months when the 100 µg dose was utilized (85). In general, 95% of the volunteers seroconverted and 86% recognized the native protein of the sporozoite. The C peptide was less immunogenic than N and R Furthermore, Montanide ISA 51 showed a better anti-sporozoite response than Montanide ISA 720 (22). Following that, in a phase II clinical trial, three LSPs were formulated with Montanide ISA-51 and applied in healthy malaria-naive and semi-immune volunteers (23). The participants received three doses of a vaccine containing a mixture of LSPs or placebo. The first dose contained N and C peptides, while the second and third contained all three peptides. After immunization, patients were challenged with sporozoites in a CHMI (23). The vaccine was safe, well tolerated, and induced sterile protection in 36.6% of the naive volunteers and 27.3% of the semi-immune volunteers against CHMI (23). Although the first immunization induced seroconversion in both groups, after the third dose, the antibodies significantly increased, mainly in the naive group (23). An increase in single-cell IFN-γ production by PBMCs during immunization was found. However, a decrease was observed after CHMI, mainly in the naive group (23). This study showed some limitations due to difficulties in the follow-up of the volunteers. Hence, a new trial (NCT 04739917) is being conducted to analyse more volunteers in malaria-endemic and nonendemic areas and evaluate other factors that may influence vaccination.

Other studies developed different LSP formulations, such as PvCS-NRC and PvNR_1_R_2_, and characterized them in preclinical tests. Both were able to induce significant immunogenicity and antigenicity in immunized mice. Furthermore, they can recognize and block sporozoite invasion *in vitro* (86, 87).

### 3.3 PvTRAP

Thrombospondin‐related anonymous protein (TRAP), also known as surface protein-2 (SSP-2), is a transmembrane protein characterized by an N-terminal hydrophobic sequence (domain I); an A-domain (domain II); a thrombospondin type 1 repeat (TSR) (domain III); a repeat region (domain IV), variable among different *Plasmodium* spp; a hydrophobic transmembrane domain (domain V); and a cytoplasmic tail region (88). PvTRAP is expressed in sporozoite micronemes and translocates to the sporozoite surface during hepatocyte invasion since it is required for sporozoite motility and interaction with host cells. Additionally, it is an essential ligand during salivary gland invasion (71, 89). This protein was related to the induction of the T-cell response and high levels of sterile protection against malaria in animal models and humans (90–92).

Castellanos et al. (93), who were interested in elucidating the immunogenicity and protective efficacy of PvTRAP, conducted a study in BALB/c mice and *Aotus* monkeys utilizing PvTRAP-derived LSP. The peptide was designed to contain the region II motif of the N-terminal, which is important for the interaction with host cells (94). This peptide was utilized with Freund’s adjuvant in mouse immunization and Montanide ISA 720 or Freund’s in monkeys, and it was immunogenic in both species (93). Specific antibodies were higher and dose-dependent in mice after the first immunization. In contrast, in monkeys, significant boosting doses were necessary, and a significant cross-reactivity with the parasite was observed with Freund’s adjuvant alone. However, the production of IFN-γ was not significant in either formulation (93).

With the same goal, Bauza et al. (95) developed two new recombinant vectors expressing PvTRAP, the chimpanzee adenovirus ChAd63 and modified vaccinia virus Ankara (MVA), and tested their potential as vaccines. They applied a heterologous prime-boost system using ChAd63-PvTRAP, followed by MVA-PvTRAP. High induction of antibodies and T-cell responses in immunized mice was found (95). The vaccine efficacy and protection were assessed utilizing infectious transgenic *P. berghei* expressing PvTRAP in rodents, which showed that both CD8+ T cells and IgG antibodies could mediate protection against malaria (95).

Bacteriophage Qβ VLPs conjugated to PvTRAP peptides were applied to identify potential B-cell epitopes with protective efficacy (96). Qβ VLPs are noninfectious particles that, when used as immunogens, can be captured and processed by APCs and presented by MHC-I and MHC-II to T helper and T cytotoxic lymphocytes, enhancing the immune response (97). In this system, a synthetic peptide is chemically coupled by a terminal cysteine residue to the Qβ VLPs. Peptides were chosen by screening sera from TRAP-vaccinated mice for immunogenic peptides or exploiting sporozoite invasion protein conservation regions, which were coupled to the Qβ platform (96). Mice were vaccinated with these formulations, and a malaria sporozoite challenge was performed to assess the protective efficacy of these epitopes (96). Four new epitopes were discovered to confer partial protection in mice in the Qβ system. Furthermore, they could inhibit sporozoite invasion *in vitro* and were recognized by antisera monoclonal antibodies from immunized mice (96). The antibodies against the conserved region are neutralizing, but just one, called TRSP, was demonstrated to have protective efficacy. Additionally, this study investigated the immune interference between peptides and showed that this is the principal challenge regarding the manufacture of a multiantigen vaccine (96). Despite the low protective efficacy of the peptides identified, this study was groundbreaking by showing the potential of Qβ-VLPs as a system to identify novel epitopes. (98) The potential of microcrystalline tyrosine (MCT) as an adjuvant in combination with PvTRAP VLPs was evaluated as a vaccine candidate in mice (98). The PvTRAP was coupled with VLPs derived from the cucumber mosaic virus fused to a universal T-cell epitope of the tetanus toxin (CMVtt) (98). A challenge was performed with *P. berghei* transgenic plants expressing PvTRAP to measure the protective capacity of these different formulations. After vaccination, the group receiving PvTRAP-CMVtt with MCT developed the highest, earlier, and more lasting antibody response than the other groups. IgG subsets showed a dominance of IgG1 response in all formulations (98). A significant induction of IgG2a, IgG2b, IFN-γ, and TNF-α production was found when MCT was utilized as an adjuvant. Moreover, this formulation showed significant protective efficacy by delaying parasitemia but no sterile protection (98).

Recombinant PvTRAP associated with three different adjuvants, NLX, CpG-ODN and 2-O-deacylated monophosphoryl lipid A (MPL), was inoculated individually or mixed and compared with CFA/IFA in mice (99). After immunization, an increase in the antibody response, especially IgG2b and IgG2c, was observed in all formulations compared to rPvTRAP alone (99). Even though all groups immunized with PvTRAP plus an adjuvant had an increase in IgG2b, IgG2c and IFN-γ, the group that received the mix of adjuvants had the highest levels of those as well as antibodies with greater avidity, which were persistent up to 180 days (99). In contrast, low levels of IL-10 and no production of IL-4 were detected in any group. These results indicate a Th1 response, which is important to eliminate intracellular parasites at the *P. vivax* liver stage; however, it is still necessary to test the efficacy of this vaccine in challenge models (99).

PvTRAP is immunogenic in natural infections since naturally acquired antibodies to recombinant PvTRAP were detected in exposed individuals from the Brazilian Amazon (100), Thailand (101), Iran (102), Afghanistan (102) and Pakistan (102).A positive correlation between the IgG3 response and longer times to the last malaria episode was found, suggesting that this subclass could be related to protection (100). These findings corroborate those from Nazeri et al. (102), who found an association of IgG1 and IgG3 antibody responses with high avidity against PvTRAP. Understanding the antibody response to a vaccine candidate in exposed individuals might help to find better candidates (102).

### 3.4 PvCelTOS

Cell-traversal protein for ookinetes and sporozoites (CelTOS) is an essential protein for the parasite to pass through cells during its life cycle through the disruption of the membrane. It binds directly on the cytosolic face of the plasma membrane, creating pores for the parasite’s exit (103). PvCelTOS was found to be highly conserved worldwide (104). A polypeptide of approximately 19 kDa was produced in *E. coli*, and 30% of patients infected with *P. vivax* had specific antibodies to recombinant PvCelTOS (105). To recognize potential B and T-cell epitopes, peptides were analysed, demonstrating that all peptides have regions with potential B-cell epitopes (105). A study carried out by Alves et al. (104) used 4 different vaccine platforms to immunize CD-1 and BALB/c mice: 1) the recombinant chimpanzee adenoviral vector (ChAd63) expressing PvCelTOS (Ad); 2) the recombinant MVA vector expressing PvCelTOS (MVA); 3) PvCelTOS conjugated to bacteriophage Qβ virus-like particles (VLPs); and 4) the PvCelTOS protein produced in eukaryotic HEK293T cells. First, ChAd63 was given intramuscularly using the Matrix-M adjuvant; the other three platforms were delivered 8 weeks later to boost responses. The highest antibody titer was after boosting with VLPs and protein (104). However, none of the platforms were able to generate cellular protection, which was assessed based on IL-2, TNF-α, and IFN-γ (104).

### 3.5 Multiantigen PEV

A vaccine targeting multiple antigens could be more effective than a single one. However, antigenic interference can prejudice immune recognition, so this fact needs to be considered when developing multiple antigen vaccines. To verify the efficacy of multiple vaccines, Atcheson et al. (106) developed a formulation combining Rv21 with viral vector TRAP, in which TRAP was expressed by recombinant adenovirus ChAd63 or modified vaccinia virus (MVA). This formulation was tested without or with AddaVax or Matrix-M adjuvants. A chimeric *P. berghei* sporozoite expressing both PvCSP and PvTRAP was used as a challenge model to assess the protective efficacy (106). The results showed 100% sterile protection, with or without adjuvant, when mice were immunized with both antigen combinations at low doses, which was not observed when they were administered alone (106). This response may be related to a high titer antibody and CD8+ T-cell response against PvCSP and PvTRAP, which can inhibit hepatocyte invasion and induce the elimination of hepatocytes infected by cytotoxic T cells. Therefore, this study showed that the combination of Rv21 and MVA-TRAP, in coadministration, improves immunity and increases protective efficacy against malaria.

### 3.6 Perspectives on PEV

Many strategies have been used to obtain an effective vaccine capable of interrupting the preerythrocytic stage. One of the main vaccines uses the irradiated whole parasite. However, due to the limitations of cell culture and sporozoite obtention, subunit vaccines are more advantageous. Among the main strategies for the development of subunit vaccines are the use of T and B-cell epitopes, viral vectors, viral particles, and multiple antigens or alleles. The most advanced studies use the PvCSP protein. To date, candidates that have reached the clinical stage have not conferred satisfactory efficacy, indicating the need for further studies and new technologies to obtain an ideal candidate. Advances in the development of vaccines targeting *P. falciparum*, such as RTS, S and R21, indicate that CSV-S, S and Rv21 vaccines targeting *P. vivax* have great potential since they showed strong responses in preclinical studies. Multiallelic vaccines targeting the three variants of PvCSP are a strategy that may help overcome the limitations imposed by the high polymorphism of *P. vivax*. However, these formulations have not yet been evaluated in clinical trials, which may indicate the future of these vaccines. PvTRAP-derived vaccines have also been used in different formulations. When expressed in viral vectors, they have the ability to induce a high antibody response and reduce parasitemia. However, more studies are needed, especially in the clinical phase, to evaluate the efficacy of these formulations. Another candidate is PvCelTOS, which is also expressed in the sexual stages of *P. vivax*, and it has highly conserved amino acid regions that are accessible to the immune system. Among the candidates presented, the multigenic vaccine that combines PvCSP (Rv21) and PvTRAP (MVA-TRAP) has high potential, since it was the only formulation that was able to induce sterile protection in animal models. This formulation will be evaluated in the clinical phase in the coming years, which could elucidate the potential of this vaccine. New technologies, such as mRNA vaccines, might bring better results; however, it is matter of testing whether known antigens would be the best on this kind of formulation or new ones need to be discovered. One of the main difficulties in eliminating *P. vivax* is the formation of latent hypnozoites. PEV may reduce the number of hypnozoites; however, the ideal would be a vaccine candidate targeting this latent form. In this sense, it is necessary to develop new studies and strategies aimed at better understanding the dormancy process and how to interrupt it.

Finally, based on what is known about PEVs, it is possible to conclude that a single antigen vaccine will not be able to induce sterile protection. Therefore, it is suggested that preerythrocytic stage antigens be combined with antigens from other stages to achieve higher efficacy. For this, it is important to identify for each antigen the most conserved and immunogenic epitopes and verify the best antigen combination and formulation.

## 4 Blood-stage vaccine

**Figure 3:**
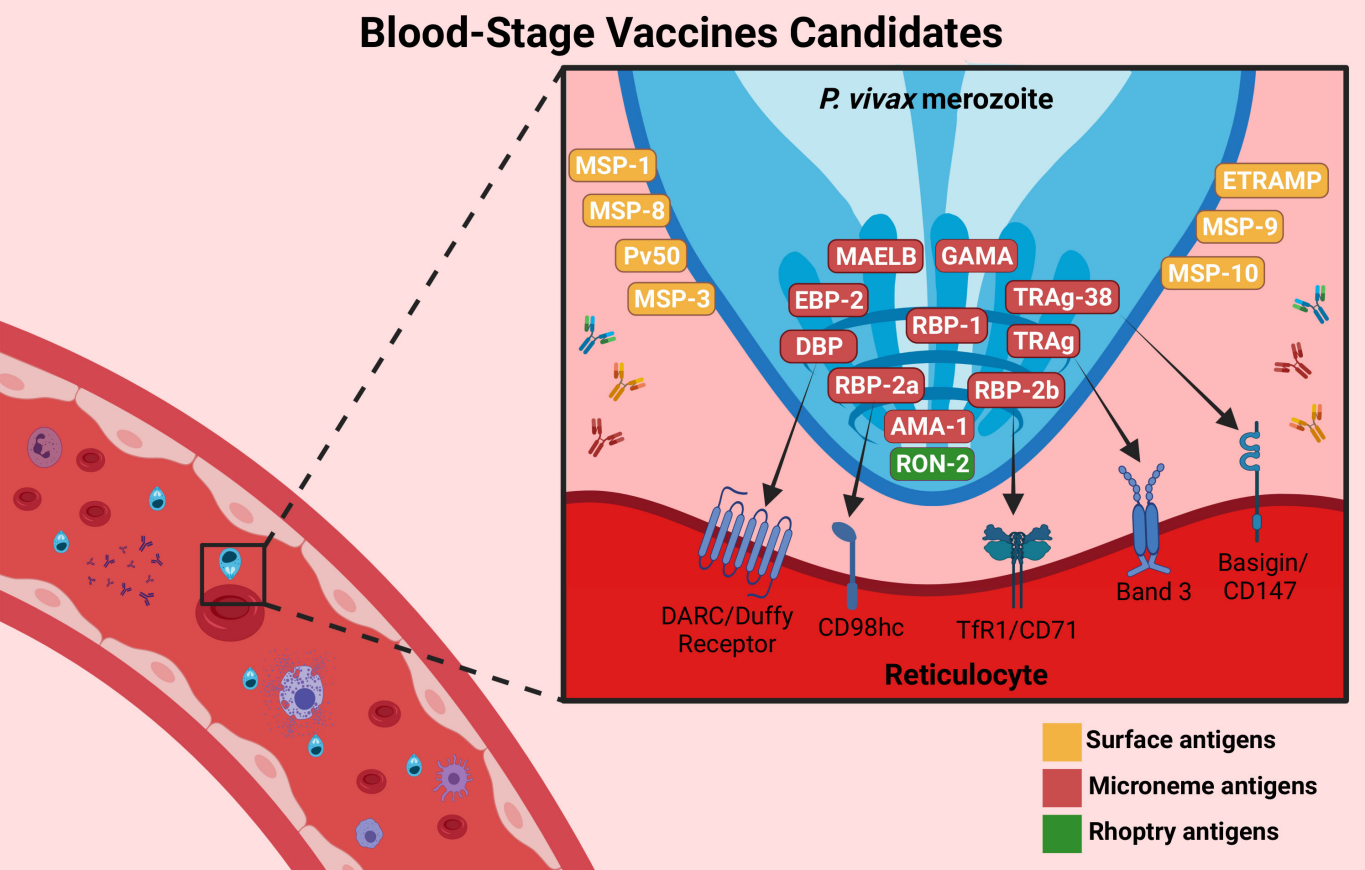
Blood-stage vaccine targets. BV candidates are important proteins involved in the invasion of *P. vivax* into reticulocytes. The main candidates are PvDBP-II (which interacts with the DARC/Duffy receptor), PvRBP-2b (which targets the TfR1/CD78 receptor), PvAMA-1, and the PvMSP family, which are involved in the invasion process. Others were recently described, such as GAMA, PvTRAg (which targets the Band 3 receptor or Basigin/CD147 receptor, such as TRAg-38), MAELB, ETRAMP, RON2 and EBP-2. They are expressed on the surface (yellow), microneme (red) or rhoptry (green) of the parasite. Antibodies against these proteins (represented by **“**Y shaped**”** on the figure) could inhibit the interaction with the reticulocyte receptors, reducing parasitemia and preventing disease symptoms. Created with BioRender.com.

Blood-stage infection is the sole cause of symptoms of malaria. The asexual blood stage involves merozoites, early and late trophozoites, and schizonts. The majority of antigens studied in BVs are expressed in merozoites and schizonts. Merozoites are the free form of *Plasmodium* parasites during the blood cycle and are therefore more exposed to the host immune system. In addition, the majority of antigens expressed during the intraerythrocytic cycle are encoded by multigene families and are very polymorphic. *P. vivax* merozoites infect reticulocytes exclusively (107). The presence of these antigens in the blood stimulates T cells and B cells to produce immune responses (108). Thus, a BV should block parasite growth, prevent disease and death, and reduce the density of parasites in the blood (parasitemia and gametocytemia) and the transmission of *P. vivax* (67). Today, the leading candidate antigen for this stage is DBPII, but other candidates, such as the RBP family, MSP family, and AMA-1, have been promising (109–111). The BV candidates are illustrated in **Figure 3**.

### 4.1 Merozoite surface antigens

#### 4.1.1 PvMSP family

The merozoite surface protein (MSP) family contains numerous blood-stage vaccine candidates. These proteins are GPI-anchored, mediate invasion into host cells and are essential to the parasite’s life cycle (112). During the last decade, several studies have investigated the potential of MSP as a vaccine candidate against malaria vivax. They showed that the antigen derived from PvMSP induces a high cellular and humoral immune response, which was associated with a reduced risk of malaria infection (113).

The main protein studied is MSP-1 (180 to 230 kDa), which is located on the merozoite surface and plays a key role in erythrocyte invasion. Several proteolytic cleavage steps occur during RBC invasion, generating diverse MSP-1 fragments. Of these, the C-terminal MSP-1_42_ fragment remains on the merozoite surface and is attached through a GPI anchor. After processing, two fragments are produced, MSP-1_33_, corresponding to the N-terminal region, and MSP-1_19_, corresponding to the C-terminal region (114, 115). MSP-1_33_ is also cleaved into two other fragments, MSP-1_14_ and MSP-1_20_. All these fragments were used in different vaccine formulations and have demonstrated a protective role in diverse animal models immunized by induction of high specific-antibody and cytokine responses and reduction of parasitemia, dependent on the adjuvant used (116–119). The most studied PvMSP-1 vaccine candidate is the 19 kDa C-terminal region, despite all being immunogenic. PvMSP-1_19_ is highly immunogenic during natural infection in individuals living in diverse malaria-endemic regions (120–122). PvMSP-1 has a homology region with Pf190 L, an immunogenic region of *P. falciparum* MSP-1 called Pv200. When utilized in vaccine formulation, it is recognized by different mouse strains and induces an immune response that is boosted following natural infection (123). Furthermore, Pv200 L is naturally immunogenic, and the presence of antibodies against Pv200 L was correlated with the number of previous infections in Brazil and Colombia (124, 125).

Cunha et al. (126) compared the immunogenicity of recombinant proteins produced in different bacterial vectors. Among the candidates analysed, His6-MSP-1_19_ and His6-MSP-1_19_-PADRE were better recognized by antibodies from several individuals exposed to *P. vivax*, inducing specific serum antibodies against MSP-1_19_. The PADRE epitope did not alter the recognition of this recombinant protein by human antibodies. In addition, the antibody immune response was dependent on the adjuvant formulations utilized (126). Rosa et al. (127) showed that the presence of PADRE epitope in vaccine formulation could significantly improve the immune response when adjuvants such as Quil A, CpG ODN 1826, MPL/TDM or MPL/TDM/CWS that are not as strong as CFA/IFA are employed. Rosa et al. (128) proved that His6MSP-1_19_ fused with two T-helper epitopes (PADRE-epitope and DYDVVYLKPLAGMYK-epitope) was highly immunogenic in *Callithrix jacchus* but only when administered with incomplete Freund’s adjuvant.

Dobrescu et al. (129) generated transgenic *P. berghei* lines expressing PvMSP-1_19_ and utilized them to challenge immunized mice with vaccine formulations based on PvMSP-1_19_ or PvMSP-1_42_. Despite the induction of high titers of specific antibodies and a balanced inflammatory process in immunized mice, the protective effects of vaccination were observed only later in the course of infection and were not sufficient to control initial parasitemia (129). However, this immune response protected immunized mice from death (129).

Fonseca et al. (130) designed a recombinant modular chimera based on PvMSP-1 (PvRMC-MSP1), including the five most promiscuous T-cell epitopes, to enhance the immunogenicity of PvMSP-1_19_ with Montanide ISA 51 in a vaccine formulation. This vaccine induced high cytophilic antibody responses in both BALB/c and C57BL/6 immunized mice (130). These antibodies recognized the native protein on the surface of merozoites. PvRMC-MSP1 elicits both CD4+ and CD8+ T-cellcell responses against MSP-1_19_, which are related to protection (130).

Sheikh et al. (131) developed a plasmid DNA vaccine encoding a 42 kDa fragment of PvMSP-1. The immunogenicity of this vaccine was investigated by priming BALB/c mice either with the PvMSP-1_42_ DNA plasmid or with recombinant PvMSP-1_42_ protein and boosting with recombinant PvMSP-1_42_ protein (131). The best immune response was induced by prime boosting with recombinant protein, which resulted in higher antibody and cytokine responses than the control and DNA alone, suggesting that the recombinant protein is essential for an improved immune response (131). Kim et al. (132) developed a vaccine formulation comprising attenuated Korea vaccinia virus (KVAC103) expressing the 33 kDa fragment of PvMSP-1. A low cellular response and a strong antibody response were obtained in immunized mice (132).

The paralogue protein of PvMSP-1, called PvMSP-1P, has also been investigated as a vaccine candidate. This protein is very similar in genetic structure to PvMSP-1 but has double epidermal growth factor (EGF)-like domains at the C-terminus. It demonstrated high immunogenicity, principally of the C-terminal region (133). Anti-PvMSP-1P_19_ human serum had significant inhibitory effects on erythrocyte binding *in an in vitro* assay (133). Min et al. (61) showed that the PvMSP-1P antigen induces a long-lasting humoral response in natural *P. vivax* infections, which was maintained for 9 months after recovery and associated with the presence of PvMSP-1P_19_-specific MBCs during the same period. Furthermore, Han et al. (115) showed that PvMSP-1P plays an important role in parasite adherence and host cell invasion. Antibodies against PvMSP-1P have a strong inhibitory effect on reticulocyte invasion, suggesting a possible role in the Duffy-negative invasion pathway (115). Han et al. (134) found two monoclonal antibodies that are able to inhibit erythrocyte binding and parasite invasion, suggesting novel epitope candidates for a subunit vaccine.

Another MSP family investigated as a vaccine candidate is MSP-3, a multigenic family located on the surface of mature schizonts and merozoites that appears to interact with the surface of erythrocytes (135). The most studied members of this family include PvMSP-3α and PvMSP-3β. These proteins are related to *P. knowlesi* and *P. falciparum* MSP-3 proteins, which were demonstrated to be immunogenic in animals and contain conserved regions that induce antibodies that block merozoite invasion (136, 137). A serological analysis performed with individuals naturally exposed to malaria vivax in the Brazilian Amazon found that PvMSP-3α is a target of the immune response, inducing a high titer of IgG antibodies, which appears to be related to protective immunity (138). Several linear B-cell epitopes were predicted in the PvMSP-3α sequence (138). The presence of antibodies against PvMSP-3α block II and the PvMSP-9 N-terminal region was associated with reducing the burden of *P. vivax* malaria and protecting against clinical disease (139). Oyong et al. (140) demonstrated that PvMSP-3α antibodies acquired during *P. vivax* infection facilitated complement fixation. Mourão et al. (121) discussed that despite the natural polymorphism of PvMSP-3α_359-798,_ it should be considered in vaccine development since specific antibodies to this antigen were observed in infected patients with limited exposure to malaria.

Bitencourt et al. (141) evaluated the antigenicity and immunogenicity of vaccine formulations based on recombinant PvMSP-3α and PvMSP-3β. A higher percentage of individuals living in endemic areas of natural infection in Brazil had antibodies against PvMSP-3α (68.2%) and PvMSP-3β (79.1%) (141). After mouse immunization, PvMSP-3β induced a humoral immune response even without any adjuvant formulation, while PvMSP-3α did not. When administered adjuvants (Alum, Quil A, TiterMax, IFA, and the TLR-5 or TLR-9 agonists FliC or CPG ODN 1826, respectively), a high induction of IgG antibodies was observed (141).

Another member of the MSP family investigated is MSP-8, which contains a GPI-anchor region and two epidermal growth factor (EGF)-like domains at the C-terminus that are important targets of protective immunity (142). PvMSP-8 induced high humoral and cellular immune responses in patients with *P. vivax* infection (143). PvMSP-8 was able to induce a long-lasting humoral immune response, since high specific antibody and MBC responses against this antigen were observed in individuals who acquired a natural *P. vivax* infection (65).

*P. vivax* MSP-9 has also been investigated as a potential vaccine candidate. It is a hydrophilic protein with a signal peptide, a cluster of four cysteines, a conserved N-terminal domain, and a C-terminal region containing blocks of species-specific tandem repeats (144). This protein is highly antigenic and immunogenic, contains several T-cell epitopes and is associated with a naturally acquired immune response (139, 145–147). Monoclonal antibodies against PvMSP-9 inhibited the parasite invasion of erythrocytes (135).

Silva et al. (30) identified a highly immunogenic linear B-cell epitope at the C-terminal portion of domain R2 of PvMSP-9, called PvMSP-9_E795-A808_. This peptide is naturally immunogenic and is recognized by IgG antibodies from individuals living in malaria-endemic areas (30). This epitope improved the immune response against a recombinant protein that contains the entire repetitive region of MSP9 (PvMSP9-RIRII) (30). The specific IgG1 against the epitope was associated with protection parameters (30). Silva et al. (148) tested this epitope’s immunogenicity using three synthetic peptides, with the sequence PvMSP-9_E795-A808_ alone or linked to the PvMSP-9_A443-K456_ T-cell epitope or the tetanus toxin universal T-cell epitope (TTRII), in BALB/c mice immunization. Both epitopes elicited specific IgG antibodies, mainly IgG1 and IgG2, to recognize the native parasite protein (148). The humoral immune response was improved only when PvMSP-9_E795-A808_ linked to the T-cell epitope TTRII was utilized, showing an important role of T-cell epitopes in improving immunity in subunit-based vaccines (148). Soares et al. (66) showed the induction of short‐lived antibodies but long-lived MBC responses to PvMSP-9_E795-A808_ in subjects exposed to declining malaria transmission in Brazil, suggesting a long-lasting immune response.

PvMSP-10 has also been investigated (149). This protein binds selectively to reticulocytes, which could be important to parasite invasion (150). Two peptides are involved and could be utilized in immunization trials since these peptides contain low polymorphism and epitopes of B and T cells and could inhibit binding to reticulocytes (150). A recombinant protein derived from PvMSP-10 when utilized in vaccine formulations with Freund’s, Montanide ISA720, or aluminum hydroxide as adjuvants in *Aotus* spp. Induced a strong immune response (151). The produced antibodies recognized the native protein in the late schizont stage (151). However, none of the formulations protected the immunized monkeys in an experimental challenge with *P. vivax* VCG-1 strain asexual blood-stage parasites (151). Cheng et al. (152) reported that anti-MSP10 antibodies are recognized by serum samples from infected patients, predominantly of cytophilic IgG1 and IgG3 responses (152). The same profile of the antibody response was observed after mouse immunization. A high cytokine response was obtained, inducing a strong immune response in immunized mice and rabbits (152).

#### 4.1.2 Pv50

The other identified antigen is a hypothetical protein of 50 kDa, called Pv50 (153). This protein showed reactivity with malaria vivax-infected patient serum (153, 154). Cheng et al. (153) showed a colocalization and a stronger interaction between Pv50 and MSP1, which could be targets of multiantigenic vaccines. A vaccine was developed with recombinant Pv50 and elicited high IgG1 and IgG3 antibody titers in mice (153).

#### 4.1.3 PvEBP-2

Erythrocyte-binding protein 2 (PvEBP-2) is also related to alternative pathways of reticulocyte invasion (155–158). Studies have suggested that this activity of PvEBP-2 occurs independently of PvDBP action (157, 158). In addition, seroepidemiological studies indicate that this protein is targeted by naturally acquired immunity in natural exposures to *P. vivax* (159, 160). Anti-PvEBP-2 antibodies were related to the protection and risk reduction of clinical disease (161, 162).

#### 4.1.4 PvETRAMP

The early transcribed membrane protein (ETRAMP), expressed at the schizont and early ring stages of *Plasmodium* spp., was also suggested as a vaccine candidate. Lee et al. (163) showed that PvETRAMP-4 and PvETRAMP-11.2 were reactive to the sera of *P. vivax* malaria patients. Furthermore, the immune response was evaluated in immunized mice. A high induction of specific antibodies, mainly IgG1 and IgG2b, against PvETRAMP-4 was observed, suggesting an immunogenic potential of this candidate (163).

#### 4.1.5 PvTRAg

An important family that has been investigated as a vaccine candidate is tryptophan-rich antigens (TRAgs), belonging to the Pv-fam-a family. At least fifteen members of this group induce both humoral and cellular immune responses in *P. vivax*-exposed individuals (164). It contains several conserved T and B-cell epitopes that could be used in vaccine design (164). Some PvTRAgs could bind to erythrocytes, and this process could be inhibited by the sera of malaria-exposed patients (164). One of them is PvTRAg-26, a subcellular protein localized in the ring-stage parasite colocalized with the caveola-vesicle complex, which has an important role in parasite invasion (165). A recombinant protein derived from this antigen presented high antigenicity and immunogenicity in mice, with a Th1 and Th2 immune response (165).

### 4.2 Microneme antigen

#### 4.2.1 PvDBP

Duffy Binding Protein (DBP) is a highly polymorphic protein located in *P. vivax* merozoites. It is the central molecule necessary for the invasion of reticulocytes due to its ability to bind to the human Duffy Antigen Receptor for Chemokines (DARC/Duffy) (166, 167). PvDBP is a 140-kDa type I membrane protein consisting of four principal regions: a peptide signal sequence (region I), two cysteine-rich regions (region II and region VI), and a transmembrane domain (region VII). The principal is region II (RII), a conserved sequence responsible for erythrocyte binding (155, 166, 167). The invasion mechanism involves different sites of RII that are an important target for vaccine development (168). PvDBP was believed to be the only reticulocyte linker, and therefore, Duffy-negative individuals would be resistant to vivax malaria (167, 169) (128). However, in recent years, it has been demonstrated that Duffy-negative individuals can be infected by *P. vivax*. This is probably due to other molecules involved in reticulocyte invasion, suggesting that the invasion mechanism is much more complex than believed (170–173).

Antibodies against PvDBP showed excellent results in blocking invasion and reducing infection (174). A long-lasting MBC response against PvDBP-II variant antigens was observed among individuals who were living in low malaria endemicity (60). Naturally, acquired PvDBP-II-specific binding inhibitory antibodies (BIAbs) are related to protection against *P. vivax* in a hyperendemic region, whereas they decreased the risk of *P. vivax* infection by 55% in children with BIAbs (175). Additionally, it was related to a delay in the time to reinfection and reduction in parasitemia. The immune response of most children with BIAbs was strain-transcending, suggesting that a vaccine based on PvDBP-II may be effective against diverse *P. vivax* strains (175). Naturally, acquired strain-specific PvDBP-II antibodies were associated with higher protection against a homologous strain than a heterologous strain. A delay in reinfection was observed when antibody levels for the most common variants were combined (176). Therefore, a multiallele vaccine could provide better protection against vivax malaria. Consequently, the identification and characterization of different PvDBP polymorphisms are essential to vaccine design since they can interfere with vaccine efficacy and facilitate parasite immune evasion (177). A B-cell epitope was identified in a polymorphic region of PvDBP-II, called the DEK epitope, which targets human inhibitory anti-DBP antibodies and is related to protection (178). The sequential region of this epitope was used to create an immunogen called DEKnull, from which the polymorphic residues were removed to evaluate its immunogenicity without the influence of the strain-specific response and focusing on the immune response toward more conserved neutralizing epitopes (179). DEKnull has a similar binding activity as the native protein and induces a high titer of antibodies and antibodies that can inhibit the binding of the native protein to erythrocytes (179). These outcomes showed that a more conserved epitope region could provide protection against malaria vivax and are potent in inducing strain transcending immunity (179). These outcomes motivated the design of DEK-null-2, which contained a conserved region important to reticulocyte binding and was removed from the nonfunctional epitopes associated with strain-specific immunity in trying to obtain a better immunogenic response. After mouse immunization, the recombinant protein induced a higher BIAbs than the original protein (180). Individuals living in a malaria-endemic region from the Brazilian Amazon had a high prevalence of IgG antibodies to DEK-null-2 until six years after the exposure and a stable BIAb response. These antibodies also inhibited *P. vivax* reticulocyte invasion *ex vivo* (180). Since DEK-null-2 is a target of naturally acquired BIAbs, it could be a good vaccine candidate, inducing a strong and long-lasting immune response (180). Medeiros et al. (181) investigated the relationship between naturally acquired PvDBP-II-specific IgM and IgG antibody/BIAb activity profiles during a nine-year follow-up period. Antibody responses were compared between two alleles derived from PvDBP, Sal-1 and the DEK-null-2 strain (181). Long-term exposure to low and unstable levels of malaria vivax transmission provokes a sustained DBP-II-specific IgM response against variant-specific epitopes that is not associated with IgG neutralizing antibodies. In contrast, the IgG response against variant-specific DBP-II was poorly sustained at a low transmission period. These results suggest that IgM antibodies indicate continuous exposure to malaria, while IgG is associated with the BIAbs response (181).

The antibody repertoire might vary in accordance with the vaccine formulation. A vaccine formulated with PvDBP-RII and TLR agonists induced a greater antibody repertoire, which was also able to inhibit the interaction of PvDBP-RII with the Duffy receptor *in vitro* when compared with a stable emulsion adjuvant (182). Chen et al. (183) identified and characterized three broadly conserved epitopes of inhibitory antibodies, called 2D10, 2H2, and 2C, to provide strain-transcending immunity and critical motifs for future vaccine design. Similarly, Urusova et al. (184) found two monoclonal antibodies derived from conserved epitopes with high neutralizing and blocking activity that were not affected by polymorphism, suggesting strain-transcending immunity. Rawlinson et al. (185) obtained and cloned monoclonal antibodies from PvDBP-II-immunized human volunteers to verify their ability to inhibit the binding of PvDBP-II to DARC and their capacity to neutralize parasite invasion in an *in vitro* assay and in clinical isolates (185). One mAb called DB9 was able to inhibit the invasion of multiple strains of *P. vivax*. This epitope is located in subdomain 3 of PvDBP-II, showing an important site of inhibition (185). George et al. (186) found a mAb from subdomain 3, called 3C9, a conserved and linear epitope capable of inducing an antibody response in mice against recombinant PvDBP-II, as well as inhibition of PvDBP-II-erythrocyte binding *in vitro*. PvDBP-II contains cryptic epitopes, such as an epitope in subdomain 1 (SD1), which confer a cross-reactive immune response to *P. falciparum* VAR2CSA. While it appears to have no role in protection against *P. vivax*, antibodies to this epitope blocked the interaction between *P. falciparum* and chondroitin sulfate A (CSA) (187). However, the potential of this epitope as a vaccine candidate for Pf-VAR2CSA remains to be elucidated (188).

A vaccine based on recombinant PvDBP-II and different adjuvants (Montanide ISA720, AS02A, MF59, QS21, and Alum) was evaluated in mice, where significant IgG production was detected with all formulations (189). However, with ISA720 and AS02A, the antibody and cytokine induction were higher. These two formulations and alum showed antibodies with higher binding inhibition of PvDBP-II to erythrocytes (189). A preclinical study with *Macaca mulatta* demonstrated the safety and immunogenicity of a vaccine derived from PvDBP-II formulated with Alhydrogel, Montanide ISA 720, or AS02A (190). Animals showed a high antibody response, mainly with ISA 720 and AS02A, which were correlated with inhibiting parasite invasion. In addition, IFN-γ induction was observed (190).

Preclinical studies were performed with human adenovirus serotype 5 (HAdV5), chimpanzee adenovirus serotype 63 (ChAd63), and modified vaccinia virus Ankara (MVA) expressing PvDBP-II to assess the increase in immunity in mice and rabbits (191). It was confirmed that PvDBP-II is highly immunogenic and provokes a strong antibody response with either viral vector and antibodies to PvDBP-II recognized the native PvDBP in *P. vivax* parasites (191). A mixed-modality approach tested two strong adjuvants, Montanide ISA720 and Abisco 100, combined with viral vectors or recombinant PvDBP-II. The results showed high IgG and IFN-γ T-cell responses in both combinations, especially after three immunizations. The highest level of binding inhibition was found in the Abisco 100 formulation (191).

A phase I clinical trial performed with healthy UK adults evaluated the formulations ChAd63 and MVA encoding PvDBP-II from the *P. vivax* Sal1 strain in a heterologous prime-boost immunization using an 8-week interval (25). This vaccine was safe and induced a binding-inhibitory antigen-specific antibody response. A high B-cell antibody and memory response was observed after MVA boosting. The majority of IgG responses were IgG1 and IgG3 subtypes (25). Additionally, an efficient IFN-γ T-cell response was found, suggesting an important role of this cell in the acquisition of malaria vivax immunity. In addition, a binding-inhibition assay with a different allele of PvDBP-II, the HMP013 Indian strain of *P. vivax*, resulted in fifty percent binding inhibition, suggesting strain-transcending immunity (25). These outcomes support the utilization of this platform in phase II clinical trials and CHMI. Similarly, another vaccine phase I clinical trial was carried out with a recombinant PvDBP-II and glucopyranosyl lipid adjuvant-stable emulsion (GLA-SE) (26). The formulation was utilized to immunize healthy Indian male adults, with 10, 25, and 50 μg doses, and was demonstrated to be safe and well tolerated (26). In addition, a strain transcending immunity was observed through an antibody response against different *P. vivax* strains (26).

#### 4.2.2 PvRBP family

The *P. vivax* reticulocyte binding protein (PvRBP) family includes important members that mediate reticulocyte invasion (46). The *P. vivax* Salvador-I genome has 11 members, five full‐length genes (*pvrbp1a*, *pvrbp1b*, *pvrbp2a*, *pvrbp2b*, and *pvrbp2c*), three partial genes (*pvrbp1p1*, *pvrbp2p1*, and *pvrbp2p2*), and three pseudogenes (pvrbp-2d, pvrbp-2e, and *pvrbp*-3) (192, 193).

Some RBPs, localized at the apical pole of merozoites, are possibly involved in the alternative invasion pathway of Duffy-negative individuals since they can bind to erythrocytes and have reticulocyte selectivity (172, 173, 193, 194). Each protein has one specific binding site different from PvDBP. Human antibodies against some RBPs were associated with parasitemia reduction and protection against clinical malaria (194, 195). Higher levels of IgG3 to PvRBP2P1 were associated with higher complement fixing capacity (195). Naturally, acquired antibodies against PvRBP-2c- and PvRBP-1a-specific domains display high reticulocyte binding-inhibitory activity (196). Gupta et al. (197) verified that a rabbit antibody against recombinant PvRBP-1a_30_ (352 aa–599 aa) inhibits the binding of PvRBP-1a to reticulocytes in a dose-dependent manner. However, these antibodies had no activity in inhibiting *P. vivax* invasion, suggesting alternative invasion pathways. This is probably due to the considerable variation in the results obtained from the *P. vivax* invasion assay from different clinical isolates (193). A specific binding domain of PvRBP-1a, called RBP1:F8 (157 aa – 650 aa), was characterized using overlapping fragments of the recombinant protein (198). RBP1:F8 was immunogenic since it induced a high level of antibody in immunized animals, and anti-RBP:F8 antibodies blocked the interaction of RBP:F8 and erythrocytes *in vitro* (198). Naturally, acquired antibodies were present in the sera of individuals exposed to *P. vivax*, which suggests that this protein is naturally immunogenic (198).

Longley et al. (199) investigated the IgG antibody responses against *P. vivax* blood stages of asymptomatic volunteers in a low-transmission region of Thailand. Among the proteins analysed, five PvRBP family members were investigated. It was observed that the magnitudes of IgG responses to different PvRBPs are generally correlated and tend to increase with age, and asymptomatic patients have high IgG responses to PvRBP-1b (199). Antibodies to the PvRBP-2c nonbinding region were associated with child protection (199). At the same time, RBP2-P2 and RBP-1b could provide an antibody response early in life and long-lasting even in the absence of new infections (199). He et al. (200) investigated the antibody response against six recombinant PvRBPs (PvRBP-1a, PvRBP-1b, PvRBP-2a, PvRBP-2b, the PvRBP-2c nonbinding region, and PvRBP2-P2) in populations living in low malaria transmission regions of Brazil and Thailand. This study showed that the IgG response to PvRBP-1a, PvRBP-2b, and PvRBP-2cNB could be a useful immunologic marker of asymptomatic *P. vivax* infection in these regions in a broad age range and predict individuals at higher prospective risk of infection (200). In addition, it was found that antibody levels against PvRBP-2b are associated with protective immunity against clinical *P. vivax* episodes, even at low levels (200).

Gruszczyk et al. (201) identified transferrin receptor 1 (TfR1 or CD71) as an important receptor involved in PvRBP-2b reticulocyte binding and elucidated the mechanism involved in this process. Blocking *P. vivax* invasion was demonstrated in the absence of this receptor in the reticulocyte membrane. In addition, monoclonal antibodies against PvRBP-2b were able to inhibit reticulocyte binding and invasion of *P. vivax* into human cells (201). These findings suggest a new blocking site to design a vaccine against the blood stage and highlight the value of PvRBP-2b as a potential vaccine candidate.

Interested in understanding the protective function of PvRBP antibodies, Chan et al. (202) obtained and characterized monoclonal antibodies to PvRBP-2b from individuals with naturally acquired immunity to *P. vivax*. The study showed that these mAbs inhibit PvRBP-2b binding to reticulocytes by blocking TfR1-Tf complex formation and identified some epitopes involved in this process (202). Different mAbs have different inhibition mechanisms, and their combination could better block parasite invasion. Additionally, it was suggested that this strategy could lessen the impact of polymorphisms that may interfere with antibody binding and is the main obstacle for *P. vivax* vaccine development (202). The identification and characterization of different epitopes involved in parasite invasion are extremely important for designing an effective vaccine.

Chim-Ong et al. (110) examined the functional characteristics of PvRBP-2P1 as an invasion ligand of *P. vivax* and its antibody response in malaria patients. It was found that rRBP-2P1 bound selectively to reticulocytes over normocytes. Rabbit antibodies against rRBP-2P1 reduced erythrocyte binding of PvRBP2-P1 in a dose-dependent manner (110). The human natural immune response to rRBP-2P1 showed an association between higher antibody responses and lower parasite densities (110). Furthermore, the response was higher in asymptomatic carriers than in patients, indicating past exposure. The interference of human antibodies in erythrocyte binding indicates a protective role (110).

#### 4.2.3 PvAMA-1

Apical membrane antigen 1 (AMA-1) is a surface protein secreted by micronemes of all *Plasmodium* species. It can play an important role in parasite invasion into host cells in the preerythrocytic and blood-stage because it is an essential component of the moving junction on the apical pole of the parasite, forming an invasion complex with rhoptry neck protein 2 (RON-2) (203, 204). AMA-1 comprises four main regions: a pro-sequence; a rich cysteine ectodomain; a transmembrane domain; and a C-terminal cytoplasmic region (205, 206). The ectodomain contains three regions, called DI, DII, and DIII (206). While the DI region has higher genetic diversity and mutation rates, the DII is the most conserved and immunogenic region of AMA-1, and it is recognized by human antibodies after natural infection (207, 208). PvAMA-1 is a promising candidate against malaria since it can induce a strong immune response and can potentially inhibit parasite growth (209). Bioinformatics analysis demonstrated that PvAMA-1 is highly immunogenic and antigenic and has desirable vaccine characteristics, such as several epitopes that could be a proper target for vaccine development (210). Bueno et al. (109) demonstrated that PvAMA-1 in a vaccine formulation modulated dendritic cell maturation by upregulating antigen-presenting molecules on the surface of the cells. Moreover, it was observed that cytokine responses were associated with clinical protection (109). In addition, Soares et al. (62) and Soares et al. (66) showed that uncomplicated vivax malaria produces short‐lived antibodies but long-lived MBC responses to PvAMA‐1 in subjects exposed to declining malaria transmission in the Amazon. This finding suggests that populations of areas with declining transmission can produce and maintain potentially protective antibodies. The presence of these MBCs could determine whether patients re-exposed to the same strain would develop a patent blood-stage infection (62).

Gentil et al. (211) designed different recombinant proteins derived from PvAMA-1 DII and evaluated its immunogenicity by immunizing BALB/c mice with different adjuvant formulations. The results showed that PvAMA-1 was immunogenic in all formulations tested. However, a better IgG response was observed with the adjuvants Quil A, TLR9 agonist CPG-ODN and TiterMax (211). Antibodies against PvAMA-1 DII were able to recognize native AMA-1 on the merozoite surface from infected patients (211). Similarly, Vicentin et al. (212) expressed PvAMA-1 in *Pichia pastoris* and compared vaccine formulations by mouse immunization. Quil A and IFA as adjuvants induced higher antibody and more balanced Th1/Th2 responses than MPLA or alum. Antibodies against PvAMA-1 have an invasion inhibitory role against diverse *P. vivax* strains (212). Someabozorg et al. (213) demonstrated that naloxone (NLX) alone as an adjuvant in a vaccine formulation with recombinant PvAMA-1 cannot induce a good immune response. However, in combination with another adjuvant, such as IFA, a balanced Th1/Th2 response was obtained (213).

Bouillet et al. (214) demonstrated that PvAMA-1 in an adenovirus system could induce long-lasting specific antibodies, with IgG1 and IgG2a production, and a strong and durable T-cell response to prime-boost vaccination with Ad5PvAMA-1/Montanide ISA720. The concurrent induction of B and T cells against AMA-1 could be significant in neutralizing *P. vivax* infection (214). Salavatifar et al. (215) demonstrated that PvAMA-1 expressed in *E. coli* could induce a long-lasting humoral immune response in immunized mice with Freund’s adjuvant. A higher level of IgG2b and IgG1 production was observed, and a balanced Th1/Th2 response persisted up to one year after the first immunization (215). Antibodies against recombinant AMA-1 recognized the native antigen on the *P. vivax* parasite (215).

Bueno et al. (216) identified a highly antigenic linear B-cell epitope of PvAMA-1 DII. Antibodies to PvAMA-1 are associated with antibody responses to DII in individuals naturally exposed to malaria, with a predominance of IgG1 and IgG3 subtypes (216). The linear epitope at residues 290-307 aa of PvAMA1-DII was recognized by 58.3% of the individuals who had antibodies to PvAMA1-DII, suggesting that a specific antibody against this epitope is produced during natural infection (216).

Several polymorphisms in parasite antigens are the major challenge of vaccine development against malaria vivax (217). Some studies have investigated the presence and influence of the PvAMA-1 polymorphism in the immune response since immunity against only one allele can induce strain-specific immunity (218–220). These polymorphisms could facilitate parasite evasion of vaccine-induced antibodies since they cannot confer protection against different parasite strains (217). Several polymorphisms were observed around binding interfaces of PvAMA-1, suggesting immune pressure in these regions (220). Conserved and low genetic diversity regions could be promising targets for vaccines (220). Additionally, it is important to consider that the immune response differs according to each polymorphism (218). Bittencourt et al. (218) demonstrated that some Brazilian haplotypes, which are variable on B-cell epitopes, can induce cross-reactivity immunity against different alleles in the same population, conferring protection against different strains. From the same perspective, França et al. (219) showed that some Brazilian polymorphisms were also cross-reactive against foreign variants. Additionally, it showed the presence of common epitopes between them and induction of strain-transcendent immunity (219). These studies suggest that the combination of critical PvAMA-1 variants in a multiallelic vaccine formulation could protect against all strains distributed around the globe (218).

#### 4.2.4 GAMA

GPI-anchored micronemal antigen (GAMA) is an apical protein with an adhesive role in apicomplexan parasites. It contains two conserved regions with reticulocyte binding properties in *P. vivax*, which could be attractive to vaccine development (221). However, the antibody response against these regions was insufficient to inhibit the interaction (221). Baquero et al. (221) suggested two functional regions, CR1 and CR2, under negative selection, which could be suitable targets for the design of vaccines.

### 4.3 Rhoptry antigen

#### 4.3.1 PvRON-2

RON-2 is expressed in late schizont rhoptries and has an important role in parasite invasion of erythrocytes by the formation of moving junctions with AMA1 and parasitophorous vacuoles (222, 223). Antibodies against this complex inhibit parasite invasion, suggesting that it could be used as a potent vaccine candidate (223). López et al. (224) identified T and B-cell epitopes that could be utilized in a vaccine formulation and tested their immunogenicity, showing a low immune response. Bittencourt et al. (225) suggested that PvRON-2 induces a long-term antibody response since naturally acquired antibodies against PvRON-2_1828–2080_, the binding region to PvAMA-1, were found in exposed individuals who were infected or not infected from a malaria-endemic region of Brazil (225).

### 4.4 Multiantigen BV

In an attempt to improve the immune response to antigens and the protective efficacy of vivax malaria vaccines, some studies have investigated the immunogenicity and protective effect of multiantigenic vaccines. Multiantigen BVs are composed of more than one BV antigen and can enhance the protective effect at the blood stage. A multiantigenic vaccine composed of both PvDBP-II and PvMSP-1_19_ demonstrated high induction of humoral response in mice immunized with Montanide ISA 720 or Alhydrogel (226). The antibody response was higher than the formulation of single-antigen vaccines. No major competition between the two antigens was observed, which is important for an effective vaccine (226). Additionally, the combined vaccine showed a high inhibitory effect of PvDBP-II binding to the DARC receptor in an *in vitro* assay (226).

Rocha et al. (227) developed a chimeric recombinant protein PvAMA1_66_-MSP1_19_ in *Pichia pastoris*, which was utilized in BALB/c and C57BL/6 immunization, with adjuvant Poly (I:C) and compared to vaccination with individual proteins (227). The serum of individuals exposed to *P. vivax* has a higher antibody titer against the chimeric protein than against the single protein (227). Immunization with chimeric proteins induces a high antibody titer, such as immunization with the PvAMA-1_66_ protein alone, and improves the immune response to PvMSP-1_19_ (227). However, the antibody titer against PvMSP-1_19_ was lower in both immunizations when compared to the PvAMA-1 antibody response, suggesting competition between the presented epitopes (227). Furthermore, the antibodies recognized the native protein on the schizonts. Additionally, high cytokine induction in both mouse strains was observed (227).

Obaldia et al. (228) assessed the effect of a vaccine containing plasmid DNA and adenovirus-vectored encoding blood-stage antigens AMA1 and MSP-1_42_ in a prime/boost heterologous immunization regimen against a blood-stage challenge in *Aotus* monkeys. The results showed that this regimen was more protective than vaccines encoding only one antigen by induction of antibody titers, reduction of parasitemia levels, and higher rates of self-cure during the experiment time (228). In addition, a negative correlation was observed between antibody titers and parasitemia levels, although sterile protection was not observed, and some antigen interference was identified (228).

A chimeric recombinant protein targeting the C-terminal region of PvMSP-1_19_ and PvMSP-8, called rPvMSP8+1, was utilized as a multiantigenic vaccine in mice (229). The immunogenicity of this formulation was compared to each PvMSP-1_19_ and PvMSP-8 single vaccine (229). The specific antibodies developed against rPvMSP8+1 could recognize native protein MSP-1 and MSP-8 of *P. vivax* and *P. cynomolgi* mature schizonts, which is phylogenetically close to *P. vivax* and sustains long-term culture *in vitro* (229). Mice immunized with the multiallelic vaccine developed a higher antibody response and showed a stronger inhibition of *P. cynomolgi* growth in an *in vitro* assay than both single vaccines (229).

### 4.5 Perspectives on BV

Blood-stage vaccines have been widely investigated. However, the major limitation for the development of blood-stage vaccines against *P. vivax* is the absence of a long-term cell culture system and low parasitemia, which hinders the identification, characterization and evaluation of new candidates. Another challenge is the high polymorphism of *P. vivax* proteins, especially those that interact with reticulocytes. Among the strategies used in these vaccines are the identification of more conserved regions and the use of T and B-cell epitopes, viral particles, DNA plasmids, and recombinant viral vectors to enhance immunogenicity. The main and most studied are PvDBP-derived vaccines, which are primarily involved in reticulocyte invasion but are highly polymorphic. Despite showing promising results in the clinical phase, the existence of alternative pathways of reticulocyte invasion indicates that a single-antigen vaccine would not be fully effective. Recent studies have identified new merozoite candidates involved in this process, such as PvAMA-1, PvRBPs, PvMSPs, RON-2, PvEBP-2, PvETRAMP, GAMA, Pv50 and PvTRAg, and it is suggested that only a vaccine with multiple antigens would be able to confer sterile protection. Thus far, the best formulations developed are those that use more than one antigen in their formulation, and it is suggested that the different antigens can activate different pathways of the immune response that, when conjugated, provoke a more protective and effective response. However, these formulations have not yet been used in clinical studies, which would be fundamental to understanding the mechanisms involved and whether the protection observed in animal models is also provoked in humans. Further studies are still needed to identify the best formulations and antigen combinations that can confer such a response. Despite several ongoing studies, the immune response to each antigen, as their ability to activate immune cells or fix complement, is still not fully elucidated, so further studies are critical.

## 5 Transmission-blocking vaccine

**Figure 4:**
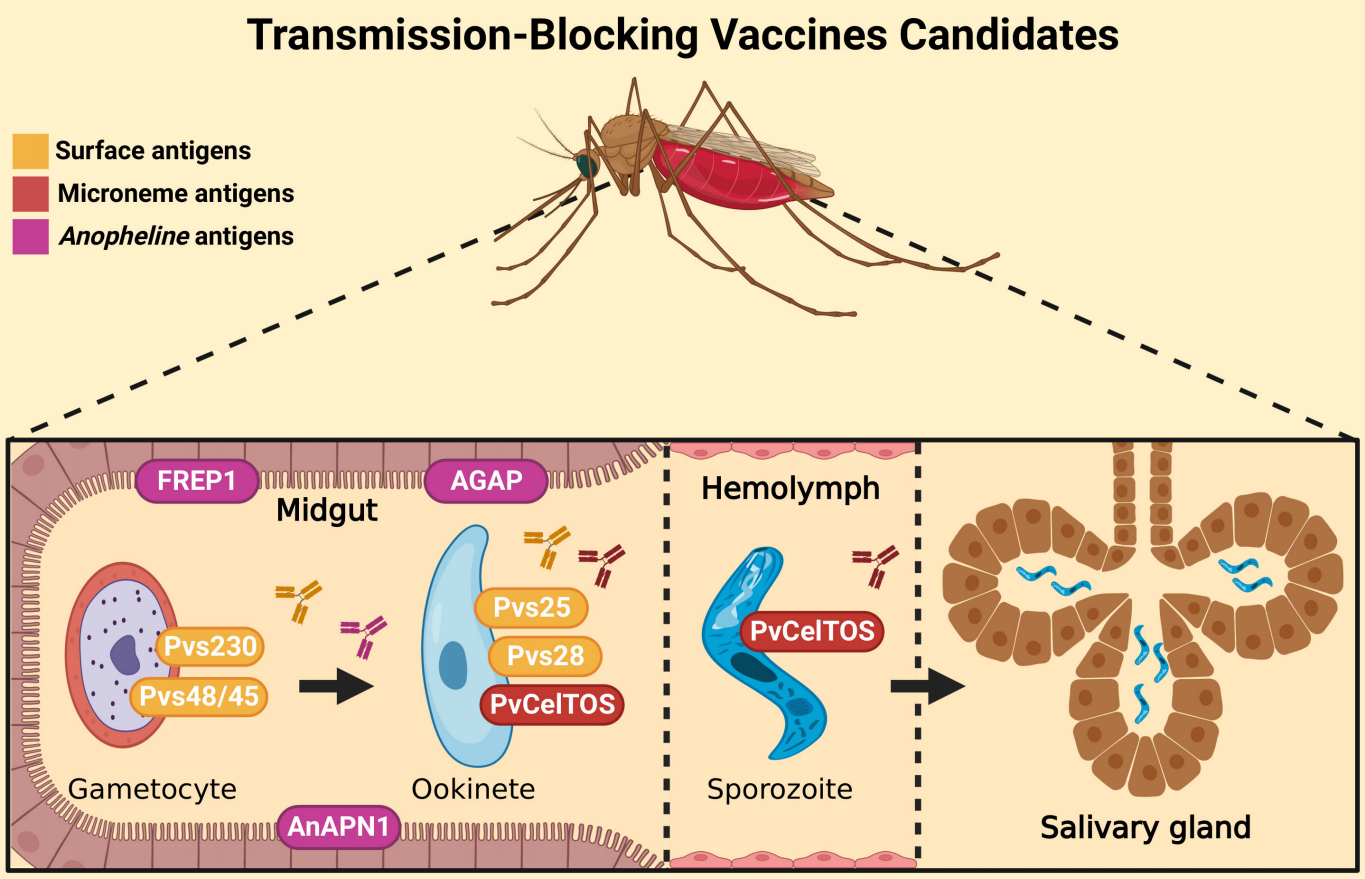
Transmission-blocking vaccine targets. TBV candidates are proteins expressed by sexual forms of the parasite. Pvs230 and Pvs48/45 are expressed on the gametocyte surface (yellow), while Pvs25 and Pvs28 (both surface antigens) and PvCelTOS (microneme antigen) are important for ookinete development. The PvCelTOS is also expressed on sporozoites microneme (red) during salivary-gland invasion. Other candidates are *Anopheline* antigens (purple), such as FREP1, AnAPN1 and AGAP, which have a role in sexual parasite form maturation. Antibodies against these proteins (represented by **“**Y shaped**”** on the figure) can interrupt the development of the following forms. Therefore, these vaccines are designed to block or reduce the transmission of the parasites. Created with BioRender.com.

TBV targets the sexual stages of the parasite, both the blood and midgut stages, within the vector mosquito. In the mosquito’s midgut, gametocytes form gametes, which fertilize to form the zygote and become the ookinete, which is the other target of TBV. Such vaccines aim to decrease or completely stop the transmission of the parasite from the intermediate host to the definitive host, preventing the maturation of the sexual phases (230). The leading candidates for TBVs in *P. vivax* malaria are the antigens Pvs25, Pvs28, Pvs45, Pvs48, and Pvs230 (**Figure 4**).

### 5.1 Pvs25

Pvs25 plays an important role in ookinete survival in the mosquito midgut, penetration in the epithelium, and transformation of the ookinete into an oocyst. A study by Miyata et al. (231) used adenovirus as a vector to assess the potential of Pvs25 to make a TBV. In a parenteral immunization test, the antibodies generated in mice reduced the number of oocysts in a membrane feeding assay by 82-99% compared to the control group. In another study, Pvs25 was linked to the cholera toxin B subunit (CTB) to assess the immunogenic potential of this antigen with that carrier molecule. When linked to CTB, Pvs25 has a significant increase in immunogenicity, reaching a transmission block of up to 98% subcutaneously and 88% intranasally in BALB/c mice (232).

Blagborough et al. (233) used a dual expression system with baculovirus to study Pvs25. Immunization of mice occurred intramuscularly and intranasally, and in both cases, the transmission block was high. The intranasal route generated a high antibody titer maintained for more than 5 months after the last immunization. In numbers, the reduction in the number of oocysts was 92.1% through the intranasal route and 83.8% through the intramuscular route. There was a predominance of IgG1, IgG2a, and IgG2b, indicating the induction of both Th1 and Th2 responses (233). Sera obtained from subcutaneously immunized rabbits exhibited a significant transmission-blocking effect (96% reduction in infection intensity, 24% reduction in prevalence) when challenged with human blood infected with *P. vivax* gametocytes using the standard membrane feeding assay (233).

Another attempt to create a plausible means of producing a TBV is the production of the Pvs25 antigen in a plant-based system. The protein was expressed in *Nicotiana benthamiana*. The vaccine candidate was tested using adjuvants, such as Abisco-100 and Alhydrogel, and the recombinant viral chimpanzee adenovirus vector expressing Pvs25 (ChAd63-Pvs25) to immunize BALB/c mice (234). The highest antibody titer was generated by a combination of adenoviral delivery, recombinant protein, and boosters with Abisco-100, reaching a 74.5% reduction in the number of oocysts in the membrane feeding assay (234).

The first phase I clinical trial in humans using Pvs25 demonstrated encouraging results, highlighting the ability of this protein to generate an immune response. Recombinant Pvs25 (Pvs25H) was used together with the adjuvant Alhydrogel. After 194 days, sera containing higher levels of antibodies generated between 20-30% reduction in the numbers of infected mosquitoes in the membrane feeding assay (235). Another study that reached phase I clinical trials used a Pvs25 protein formulated with the adjuvant Montanide ISA 51. In this case, the research was interrupted because 2 volunteers had adverse reactions. This may be due to the combination of Pvs25 and the adjuvant because Pvs25 combined with Alhydrogel was well accepted (27) but with low efficacy, as shown above.

### 5.2 Pvs28

Pvs28, like Pvs25, is present in the ookinete and is related to the survival of the ookinete in the mosquito midgut, penetration of the epithelium, and transformation of the ookinete into the oocyst. As they have redundant functions, Pvs25 and Pvs28 can be used together for a more effective vaccine, since the absence of one of them does not seem to impede oocyst maturation. Knockout of both genes can almost completely block transmission (236). Antibodies to the recombinant Pvs28 protein were able to block the development of sporozoites in the *Anopheles* vector. BALB/c mice immunized with this protein and CTB had antibodies to Pvs28 for 6 months after immunization (237). Hisaeda et al. (238) generated two recombinant proteins, Pvs25 and Pvs28, and immunized mice with different genetic backgrounds. While immunization with Pvs25 induced antibodies and T-cell proliferation in all mouse strains, Pvs28 did not induce antibodies or T-cell proliferation in C57BL/6 mice. Mouse serum against both proteins blocked parasite development into mosquitos (238).

### 5.3 Pvs48/45

Pvs48/45 is expressed on the surface of gametocytes/gametes and plays a crucial role in gametic fusion during fertilization. Arévalo-Herrera et al. (239) evaluated the immunogenicity of recombinant Pvs48/45 proteins expressed in *E. coli*. All immunized mice developed a high titer of specific antibodies and seroconverted after the first dose. In addition, *Aotus* monkeys were also immunized and seroconverted, maintaining detectable levels of antibodies for more than five months after the third dose. These antibodies were able to completely block the transmission of gametocytes to the vector in a membrane feed assay (MFA), demonstrating that the epitopes maintain their conformations (239). Tachibana et al. (240) immunized mice with DNA plasmids encoding the full-length Pvs48/45. Antibodies produced in mice recognized the parasite’s native Pvs48/45 proteins, generating a significant reduction in the number of oocysts in the mosquito intestine when combined with native serum in a membrane feeding assay.

### 5.4 Pvs230

Pvs230 is a prefertilization gametocyte antigen with low polymorphism (241). To assess the immunogenic potential of this protein, Tentokam et al. (242) conducted a study with the first domain of Pvs230 (Pvs230D1 M), using serum from patients in Brazil and Cambodia to assess seroprevalence in endemic locations in both countries (242). In Brazil, 27.1% of the participants had specific IgG for Pvs230D1 M, and in Cambodia, the seroprevalence was 26.6%. The differential immune response among human IgG subtypes was evaluated, with IgG3 and IgG1 being the most prevalent, suggesting that the immune response can be improved by complement since the two IgG isotypes most present in the study are known to fix complement (242). Tachibana et al. (243) conducted a study with DNA immunization to assess the potential of antibodies to Pv230 to block transmission using *P. vivax* samples obtained from Thailand in a membrane feeding assay. The anti-Pv230 serum significantly reduced the number of oocysts in the vector in 2 out of 3 patients. With one of the samples, however, the number of oocysts was reduced, but the infection rate was not, suggesting that a higher antibody titer is needed to clear the infection and completely block transmission.

### 5.5 Other TBV candidates

Some other antigens have also been studied, such as TBV. One of these antigens is FREP1, a protein present in the gut of *Anopheles* mosquitoes that participates in invading the ookinete through direct connection with gametocytes and ookinetes (244). Niu et al. (245) evaluated the immunogenic potential of FREP1 using Hsd: ND4 mice, which were immunized subcutaneously and boosted twice at 3-week intervals with 20 μg of FREP1 per mouse in Alhydrogel adjuvant (245). Similarly, five mice were immunized with the highly conserved portion of fibrinogen-like protein (FBG) under the same regimen for optimal prime boosting. The results showed that the anti-FREP1 antibody significantly reduced the number of oocysts of *P. vivax* per midgut more than 2-fold compared to the control serum (245).

Another protein under study is alanyl aminopeptidase N specific from the middle intestine of *Anopheles* (AnAPN1), which is highly conserved among the species of *Anopheles* and is a putative target for the invasion of ookinetes by *P. falciparum* and *P. vivax* (246). To evaluate the vaccine potential of AnAPN1, Mathias et al. (247) immunized BALB/c mice with 2 μg of AnAPN1 without adjuvant, while the control group received only IFA. There were no major differences between the regimens, and it was possible to verify that the antigen induced a good immunological response (247). The blocking activity of anti-AnAPN1 antibodies was achieved by recognizing a highly conserved epitope. This peptide maintained almost 100% similarity among all species of *Anopheles* and is a great candidate for future vaccine studies (248).

### 5.6 Perspectives on TBV

Transmission-blocking vaccines have been widely investigated in recent years. However, in natural infections, a weak immune response against these antigens is induced. Consequently, in immunization systems, many doses are needed to observe high efficacy. Another limitation is the lack of *in vitro* culture of *P. vivax*, which makes it difficult to identify antigens expressed in the early stages of the gametocyte. However, several candidates have been identified that are expressed in gametocytes and ookynetes or are present in the vector salivary gland. The antigens Pvs25/28, Pvs48/45 and Pvs230 are the most studied, Pvs25 being the only one to reach a phase I clinical trial. Even so, Pvs28, as well as Pvs48/45 and Pvs230, have shown good immunogenicity, where the specific antibodies reduce transmission by preventing maturation of the oocyst inside the mosquito. In an attempt to avoid genetic variations of antigens between *Plasmodium* species and to create a universal vaccine, the antigens of the anopheline vector are also of great interest for the production of an effective TBV, such as the already known FREP1 and AnAPN1 but also other proteins to be studied and characterized, as is the case of AGAP008138, which is exclusive to the *Anopheles* species and acts by facilitating the invasion of the oocysts of more than one species of *Plasmodium.*

Moreover, further studies are needed to identify new candidates and adjuvant formulations to improve TVB response. In addition, antigens from sexual stages should be used in combination with antigens from other stages to assess protection and efficacy.

## 6 Multistage vaccine

### 6.1 MAELB

The merozoite adhesive erythrocytic binding protein (MAEBL) was associated with protection in *Plasmodium yoelli* (249). MAEBL is a membrane protein of the erythrocyte binding protein (EBL) family. It is expressed in preerythrocytes, blood stage, and salivary glands (31). Immunoinformatic analysis showed that MAEBL antigens could be promising interspecies and inter strain malaria candidates since they have several conserved epitopes among *P. yoelli*, *P. falciparum* and *P. vivax* (31). Functional studies showed that antibodies against PyMAEBL-M2 were reactive against *P. falciparum* and *P. vivax* with significant inhibition of erythrocyte invasion of these parasites (31).

### 6.2 Multistage and multiantigen

A multistage vaccine is composed of antigens from different parasite stages. Vaccines based on antigens from different stages could block different parts of the parasite life cycle and, in theory, could be better strategies for a better vaccine. Lima et al. (250) analysed the immunogenicity of vaccine formulations composed of yPvCSP-All_FL_ (PEV candidate) and PvAMA-1 (BV candidate), alone or in combination, with Poly (I:C) as an adjuvant in BALB/c and C57BL/6 mice. The BALB/c antibody response was relatively low against PvCSP, while PvAMA-1 and the mixed vaccine were higher, which could be related to an immunodominance of the epitopes of PvAMA-1 (250). In contrast, the C57BL/6 antibody response was higher and long-lasting against both immunizations, and no difference was observed in the mixed vaccine (250). In the IgG profile, a predominance of the Th2 response was observed, and the presence of PvAMA-1 seems to improve the balance of the immune response (250). However, in the mixed vaccine, antigenic interference was observed in the cell-specific proliferative response and in cytokine secretion (250). A decrease in parasitemia was observed in both immunizations after challenge with transgenic Pb/PvCSP-VK210 sporozoites, although sterile protection was not observed (250).

PvMSP-1 (BV candidate) was utilized in a chimeric formulation fused with Pvs25 (TBV candidate) in a multiantigenic vaccine (251). PvMSP-1 improved Pvs25 immunogenicity (251). The vaccine induced a high antibody titer, blocking the transmission of *P. vivax* in the direct membrane-feeding assay and producing long-lived plasma cells (251).

## 7 Conclusion

The achievement of a vaccine capable of fighting *Plasmodium* species is one of the main objectives for combating, controlling, and eliminating malaria. The vaccines approved against *P. falciparum* have presented encouraging results, reinforcing the need for a vaccine against *P. vivax* since this is the most geographically distributed parasite. Due to numerous difficulties related to it, there is still no ideal vaccine. However, several candidates targeting different stages of the parasite life cycle have been investigated.

However, single-antigen vaccines are not able to confer sterile protection, although they contribute to the reduction of parasitemia and transmission. On the other hand, vaccines that combine more than one antigen have shown promising results but are still in the early stages. Multistage vaccines can increase human reactivity to genetically diverse populations of *Plasmodium* spp. by eliciting strain-transcending immunity. These vaccines were able to induce a better immune response and increased protection in preclinical testing. Nevertheless, the best combination of antigens, platforms, and adjuvants remains unclear, denoting the need for further studies and greater investment in research targeting *P. vivax*. Due to the huge gap in knowledge about the biology of *P. vivax*, a huge delay in vaccine development is observed. Future studies should investigate unknown proteins to identify and characterize new vaccine candidates. Vaccine candidates should induce an effective protective response. When looking for antibodies as markers for vaccine candidates, it should be important to look for functional antibodies, and it is important to look for subclasses, avidity, and the ability to fix complement and not only titers. In addition, new constructs and formulations should be evaluated and compared in preclinical and clinical studies. To this end, new technologies and platforms that already exist, such as viral particles, nanotechnology, viral vectors, and mRNA, should be used but slightly explored for this parasite. In addition, it is important to identify the most conserved regions and epitopes of *P. vivax* proteins to overcome their high polymorphism and even use them in multiantigen vaccine constructions. Immunization strategies should also be analysed, and the use of heterologous prime-boost has been very promising. New adjuvants should be investigated, their mechanism of action, and their safety for use in humans. All these aspects should be considered in an attempt to obtain candidates capable of inducing a potent cellular and humoral immune response, with a robust production of neutralizing antibodies and long-lasting memory cells. The study of the immune response in *P. vivax* natural infections is essential for the identification of new candidates. Due to several limitations, such as the absence of an *in vitro* culture system and limited access to experimental models to screen new candidates, it is crucial to develop new studies aimed at optimizing these aspects, which will have great repercussions on the development of malaria vaccines. In summary, the best way for vaccine development targeting *P. vivax* is to improve multistage vaccines, as they could disrupt the different stages of the parasite life cycle. Nevertheless, further studies are needed to identify and characterize new candidates and evaluate the best combinations of antigens, platforms, immunization systems, and vaccine formulation technologies in an attempt to achieve a safe and effective vaccine.

## Author contributions

GTSV, MM, JV, and LA wrote the final version of the manuscript. MM-S reviewed the final version. All authors read and approved the final manuscript.
